# Características de las lesiones de mancha blanca asociadas al tratamiento de ortodoncia: una revisión

**DOI:** 10.21142/2523-2754-1103-2023-168

**Published:** 2023-09-26

**Authors:** Michella Vicenza Consoli Senno, Julissa Janet Robles Ruíz

**Affiliations:** Division of orthodontic, Universidad Científica del Sur. Lima, Perú. 100070186@cientifica.edu.pe, jrobles@cientifica.edu.pe Universidad Científica del Sur Division of orthodontic Universidad Científica del Sur Lima Peru 100070186@cientifica.edu.pe jrobles@cientifica.edu.pe

**Keywords:** lesiones de manchas blancas, tratamiento de ortodoncia, ortodoncia con aparatología fija, pacientes de ortodoncia, white spot lesions, orthodontic treatment, orthodontics with fixed appliances, orthodontic patients

## Abstract

**Introducción:**

El propósito de esta revisión de literatura es proporcionar evidencia científica sobre las características de las lesiones de mancha blanca (LMB) durante el tratamiento de ortodoncia, su incidencia, prevalencia, factores de riesgo relacionados con su desarrollo, progresión y regresión; además de explicar los métodos de diagnóstico más utilizados.

**Materiales y métodos:**

Se realizó una búsqueda exhaustiva en las bases de datos de PubMed, Scopus, ScienceDirect y Embase hasta la fecha del 30 de noviembre de 2022. Se incluyeron estudios transversales que evaluaron las lesiones de manchas blancas antes y después del tratamiento de ortodoncia. Dos investigadores seleccionaron cuidadosamente los artículos evaluados y analizaron diferentes tópicos clave sobre el tema.

**Resultados:**

Se encontró que la prevalencia e incidencia de manchas blancas durante el tratamiento de ortodoncia varía ampliamente según el método de diagnóstico utilizado, el tipo de técnica ortodóntica empleada y el tiempo de tratamiento ortodóntico. La incidencia de lesiones de mancha blanca es mayor en pacientes tratados con brackets convencionales, seguido de aquellos que usaron brackets de autoligado y fue menor en pacientes que usaron alineadores. Las piezas más afectadas son los incisivos laterales y caninos maxilares a nivel del tercio gingival.

**Conclusiones:**

La incidencia de manchas blancas se asocia al tratamiento de ortodoncia, y está estrechamente relacionada con la técnica de tratamiento utilizada y el tiempo de tratamiento. Existe una regresión de las LMB durante el primer año posterior al retiro de la aparatología.

## Abstract (en)

**Introduction:**

The purpose of this literature review is to provide scientific evidence on the characteristics of white spot lesions (WSL) during orthodontic treatment, their incidence, prevalence, risk factors related to their development, progression, and regression, as well as explain the most used diagnostic methods.

**Materials and methods:**

An exhaustive search was carried out in the databases of Pubmed, Scopus, Science Direct and Embase up to the date of November 30, 2022. Cross-sectional studies that evaluated white spot lesions before and after orthodontic treatments were included. Two researchers carefully selected the articles evaluated and analyzed different key topics on the subject.

**Results:**

It was found that the prevalence and incidence of white spots during orthodontic treatment varies widely depending on the diagnostic method used, the type of orthodontic technique used and the time of orthodontic treatment. The incidence of white spot lesions is highest in patients treated with conventional braces, followed by those who used self-ligating braces, and was lowest in patients who used aligners. The most affected teeth are the upper lateral incisors and upper maxillary canines at the level of the gingival third.

**Conclusions:**

The incidence of white spots is associated with orthodontic treatment, being closely related to the treatment technique used and the treatment time. There is a regression of the LMB during the first year after removal of the appliance.

## INTRODUCCIÓN

El término “mancha blanca” (LMB) es definido por Fejerskov como “la primera señal de lesión de caries en el esmalte que se puede detectar a simple vista” y se utiliza junto con los términos “inicial” o “incipiente” ^1^. 

Las lesiones de manchas blancas son una de las principales consecuencias indeseables del tratamiento de ortodoncia con aparatología fija ^2^. Su etiología se asocia al acopio de placa en forma prolongada alrededor de la aparatología fija de ortodoncia. Las superficies discontinuas de los *brackets*, bandas y alambres dificultan las técnicas de limpieza oral convencionales y aumentan la cantidad de sitios de retención de placa en las superficies de piezas dentales que normalmente son menos susceptibles al desarrollo de caries ^3^. Además de ser originado por un proceso cariológico, las manchas blancas en la superficie del esmalte pueden estar asociadas a la existencia de factores de riesgo, como el mal cumplimiento del tratamiento de ortodoncia, una limpieza oral deficiente, un tiempo de tratamiento ortodóntico prolongado, la edad de los pacientes y las alteraciones del desarrollo durante la formación del esmalte ^2^^,^^4^.

Los factores señalados hacen que las lesiones de manchas blancas sean frecuentes en estos pacientes, aunque se ha evidenciado que su frecuencia es muy variable, del 2% al 97%. Es importante señalar que la prevalencia varía dependiendo del método de diagnóstico utilizado. Gorelick *et al*. ^6^, haciendo uso de la técnica de examen visual, constataron que el 50% de los pacientes tuvieron una o más lesiones al final del tratamiento. Mediante la técnica de fluoroscopia cuantitativa, Boersma *et al*. ^7^^,^^9^ informaron que el 97% de los sujetos tenían una o más lesiones de manchas blancas durante el tratamiento con aparatología fija. Mizrahi ^8^, en un estudio transversal, evaluó 527 pacientes antes del tratamiento de ortodoncia y 269 pacientes después del tratamiento, y halló un aumento significativo tanto en la prevalencia como en la gravedad de las lesiones con el tratamiento.

La amplia variación en la prevalencia de LMB reportada en los estudios podría deberse a diferencias en las metodologías empleadas. Sin duda, el tamaño de muestra pequeño es crucial. Asimismo, los múltiples métodos de diagnóstico, tales como inspección visual, imágenes fotográficas, métodos fluorescentes, transiluminación con fibra óptica, fluorescencia láser, entre otros, también juegan un papel importante ^10^. Otra limitación de los estudios sobre LMB es la falta de consenso en el protocolo de prevención y tratamiento en estos pacientes ^9^. 

El propósito de esta revisión de literatura es proporcionar evidencia científica sobre las características de las lesiones de mancha blanca durante el tratamiento de ortodoncia, su incidencia, prevalencia, factores de riesgo relacionados con su desarrollo, progresión y regresión, además de explicar los métodos más utilizados para su diagnóstico. 

## MATERIALES Y MÉTODOS

## Estrategia de búsqueda

Se utilizaron las bases de datos PubMed, Embase, ScienceDirect y Scopus para la recopilación de la información. Los términos de búsqueda fueron descriptores o palabras clave, como *white spot lesions*, *WSL*, *orthodontic treatment*, *fixed orthodontic treatment*, *orthodontic patients*, *during orthodontic treatment*. Las referencias fueron ordenadas mediante la búsqueda de citas Refworks para evitar duplicados (Tabla 1).

**Tabla 1:** Estrategia de búsqueda de descriptores de las diferentes bases de datos

Medline/PubMed (30/11/2022)
n = 485
("white lesion"[title/abstract] OR "white spot"[title/abstract] OR WSL [title/abstract]) AND (orthodontic[title/abstract] OR "fix orthodontic"[title/abstract])
Scopus (30/11/2022)
n = 398
TITLE-ABS-KEY ((“white lesion” OR “white spot” OR wsl) AND (orthodontic OR “fix orthodontic”)) AND (LIMIT-TO (DOCTYPE, “ar”)) AND (LIMIT-TO (SUBJAREA, “DENT”)) AND (LIMIT-TO (LANGUAGE, “English”))
Embase (30/11/2022)
n = 382
("white lesion" OR "white spot" OR WSL) AND (orthodontic OR "fix orthodontic") Filter: Research articles
ScienceDirect (30/11/2022)
n = 121
('white lesion':ti,ab,kw OR 'white spot':ti,ab,kw OR wsl:ti,ab,kw) AND (orthodontic:ti,ab,kw OR 'fix orthodontic':ti,ab,kw) AND 'article'/it AND 'article'/it AND [english]/lim

## Criterios de selección

Los criterios de inclusión fueron estudios transversales o longitudinales, ensayos clínicos, casos y controles, cohortes y comparativos. Asimismo, estudios con métodos de diagnóstico para LMB como inspección visual, imágenes fotográficas, métodos fluorescentes, transiluminación con fibra óptica o fluorescencia láser. Tratamientos de ortodoncia con aparatología (*brackets* metálicos o estéticos, y alineadores) y estudios con una muestra mínima de 30 pacientes. Se excluyeron estudios que fueran revisiones sistemáticas, revisiones de literatura, reportes de caso o series de casos, artículos de opinión, comentarios o editoriales, así como estudios en animales, estudios *in vitro* y estudios con texto incompleto (“*no full text*”). 

## Extracción de datos

**Figura 1:**
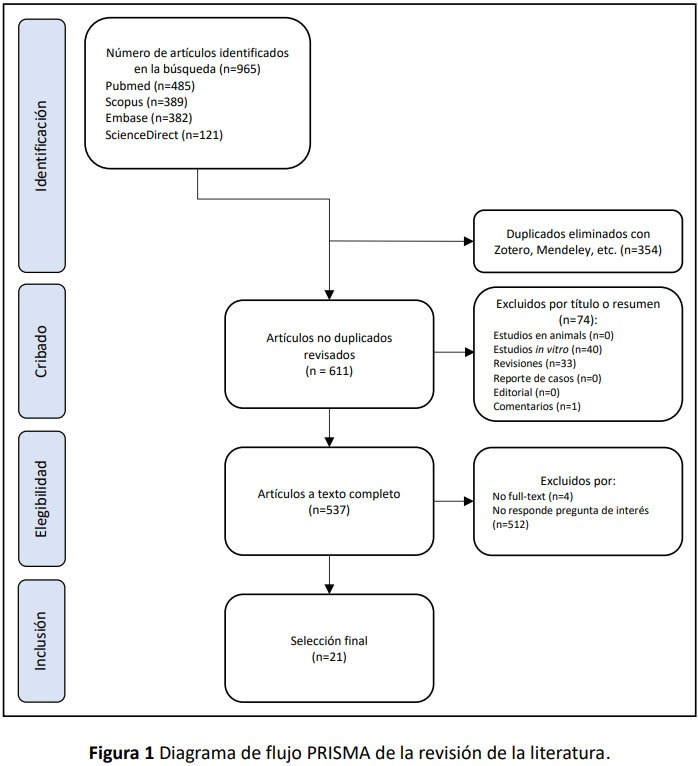
Diagrama de flujo PRISMA de la revisión de la literatura.

Los estudios seleccionados se evaluaron con base en los títulos y el resumen, según los criterios de inclusión. Esta revisión fue realizada de forma independiente por dos investigadoras. Si el artículo seleccionado no contaba con resumen, se evaluó el texto completo. En caso el artículo no cumpliera con los criterios de inclusión, fue excluido. La manera de elegir los artículos siguió el método PRISMA (Preferred Reporting Items for Systematic Reviews and Meta-Analyses) (Figura 1).

## RESULTADOS

De cada investigación se extrajo el título, autor, año, diseño de estudio, muestra, variables, valor p, instrumento de recolección de datos, dientes evaluados y resultados. El autor fue quien realizó la extracción de los resultados de interés. 

### Identificación de artículos

En la búsqueda, se identificaron 965 artículos distribuidos en PubMed (485), Scopus (389), Embase (382) y ScienceDirect (121), y se encontraron 354 duplicados con Refworks. De los 611 artículos no duplicados, se excluyeron 74 por título o resumen (40 estudios *in vitro*, 33 revisiones, 1 comentario) y quedaron 537 en la revisión a texto completo, de los cuales 512 no respondieron la pregunta de interés y 4 fueron “*no full text*”. Finalmente, fueron seleccionados 21 artículos (Figura 1).

### Características de diseños y demografía

Se incluyeron 21 artículos que cumplieron con los criterios de selección, de los cuales 12 fueron estudios transversales (57% del total de artículos seleccionados). El rango del tamaño de las muestras de los estudios incluidos osciló ente 42 y 885 pacientes, de los cuales entre 26 y 507 fueron de sexo femenino, y entre 19 y 378, de sexo masculino. En cuanto a la edad de los pacientes, esta fluctuó entre los 10 y 32 años. 

### Incidencia de lesiones de manchas blancas durante el tratamiento de ortodoncia según edad, sexo y tipo de diente de los pacientes

La incidencia de LMB durante el tratamiento de ortodoncia varía ampliamente (30%-70%) ^19^^-^^26^^,^^29^^-^^37^. Akin *et al*. ^19^ indican que se desarrolla al menos 1 nueva LMB durante el tratamiento. Por otro lado, de los 21 estudios, solo 2 concluyen que existe una mayor probabilidad de desarrollar LMB si el paciente se encuentra dentro del rango entre los 11 y 15 años ^1^^,^^29^ (Tabla 2). En cuanto al sexo, 11 de los 21 estudios determinaron que no existe una relación entre la variable mencionada y la aparición de LMB ^19^^,^^20^^,^^21^^,^^23^^-^^26^^,^^30^^,^^33^^,^^36^^,^^37^; sin embargo, 3 estudios afirmaron que el sexo masculino tiene mayor probabilidad de desarrollar nuevas LMB ^28^^,^^31^^,^^37^. 

**Tabla 2:** Incidencia de lesiones de manchas blancas durante el tratamiento de ortodoncia según rango de edad.

Ítem	Autor	No hacen referencia	No hay relación	Si hay relación	Edad	P valor
1	Akin^19^			X	11 - 14 años	0.004*
2	Albhaisi^20^		X			-
3	Almosa^21^		X			> 0.05
4	Artun^22^	X				
5	Beerens^23^	X				
6	Benkaddour^24^		X			0.83
7	Boersma^25^		X			-
8	Buschang^26^		X			0.30
9	Eltayeb^27^		X			0.78
10	Enaia^28^		X			0.06
11	Jiang^29^			X	11 - 15 años	0.042*
12	Julien^30^	X				
13	Khalaf^31^		X			> 0.05
14	Lovrov^32^	X				
15	Lucchese^33^	X				
16	Mattousch^34^	X				
17	Mizrahi^35^	X				
18	Ogaard^36^	X				
19	Richter^37^		X			0.07
20	Tufekci^38^	X				
21	Van Der Veen^39^	X				
Total	21	10	9	2		

*Significativo

Por otro lado, se encontró una mayor incidencia de LMB en la maxila que en la mandíbula ^22^^,^^26^. Según la pieza dental, se encontró mayor cantidad de lesiones en incisivos laterales superiores ^22^^,^^29^^,^^30^^,^^31^, seguidos por los caninos y premolares superiores ^24^^-^^27^, y los primeros molares inferiores ^33^^,^^35^^,^^36^^,^^39^. La zona con mayor cantidad de lesiones fue el tercio cervical ^22^^,^^31^^,^^34^. 

### Presencia de lesiones de manchas blancas según tipo de técnica ortodóntica utilizada

Se encontró que la aparición de nuevas lesiones de manchas blancas depende del tipo de técnica ortodóntica utilizada durante el tratamiento. Por ejemplo, en el caso del uso de alineadores, se encontró una incidencia entre el 1,2% ^26^ y el 6,21% ^20^, mientras que con el uso de ortodoncia fija la incidencia varía entre 8.25% y 72.9% ^20^^,^^24^^,^^26^^,^^28^^,^^30^^,^^32^^,^^33^^,^^37^, lo que nos indica que existe una mayor incidencia de lesiones de manchas blancas en pacientes con ortodoncia fija. Sin embargo, las lesiones producidas en pacientes con alineadores presentan una mayor área de lesión que en los pacientes tratados con ortodoncia fija ^20^. 

Los estudios reportan también que la técnica de ortodoncia con ligado convencional representa un mayor riesgo de desarrollar LMB (54%), a diferencia de la ortodoncia con autoligado (49%) ^19^ (p = 0,05).

### Presencia de lesiones de manchas blancas según tiempo de duración del tratamiento de ortodoncia

Todos los estudios analizados refieren que un tiempo de tratamiento mayor es directamente proporcional a una mayor probabilidad de desarrollar lesiones de manchas blancas ^19^^-^^39^. En el estudio de Lucchese *et al*. ^33^, los pacientes que tuvieron un tiempo de tratamiento de 6 meses tuvieron una incidencia del 40% de LMB y los pacientes con 12 meses de tratamiento, una del 43%. En esa línea, Tufekci *et al*. ^38^ hallaron una incidencia del 38% en los pacientes con 6 meses de tratamiento y el 46% en pacientes con 12 meses de tratamiento. En tratamientos de 18 meses de duración, Jiang *et al*. ^29^ encontraron una incidencia del 66.7% de LMB, mientras que con tratamientos que duran más de 36 meses los pacientes son 3,65 veces más propensos a desarrollar LMB ^31^.

### Prevalencia e incidencia de lesiones de manchas blancas durante el tratamiento de ortodoncia según el método de diagnóstico utilizado.

Los porcentajes de prevalencia e incidencia de lesiones de manchas blancas durante el tratamiento de ortodoncia según el método de diagnóstico utilizado son muy variables. De los 21 estudios revisados, 9 utilizaron la fotografía digital intraoral como método de diagnóstico ^19^^,^^23^^,^^24^^,^^26^^,^^28^^,^^30^^-^^32^^,^^37^, 4 hicieron uso del análisis de fluorescencia inducida por luz (FIL) ^20^^,^^25^^,^^34^^,^^39^ y 8 usaron la inspección visual ^21^^,^^22^^,^^27^^,^^29^^,^^33^^,^^35^^,^^36^^,^^38^, de los cuales 2 utilizaron el índice ICDAS ^21^^,^^27^ y 3 el índice propuesto por Gorelick ^29^^,^^33^^,^^36^.

La prevalencia de LMB antes del tratamiento utilizando el método de diagnóstico por medio de fotografías intraorales digitales varía entre el 9% y el 32% ^19^^,^^26^^,^^28^^,^^30^, y cuando el diagnóstico es por medio del análisis de fluorescencia inducida por luz todos los pacientes tenían al menos una LMB antes del tratamiento ^20^^,^^25^^,^^34^^,^^39^.

La prevalencia de lesiones de manchas blancas después del tratamiento utilizando el método de diagnóstico de inspección visual varía entre el 31,7% y el 57,7% ^21^^,^^29^; en el diagnóstico por medio de fotografías intraorales digitales, entre el 32% y el 73,3% ^23^^,^^28^^,^^30^; y en el diagnóstico por medio del análisis de FIL, entre el 31% y el 49% ^25^^,^^34^. 

### Progresión y regresión de las lesiones de manchas blancas durante el tratamiento de ortodoncia

Moniek *et al*. ^23^ mencionan que existe una regresión de las LMB durante el primer año después del retiro de la aparatología. También indican que los pacientes que presentaban lesiones de manchas blancas superficiales en el esmalte no progresaban a convertirse en lesiones cavitadas durante el primer año después del retiro de la aparatología. 

## DISCUSIÓN

Pese a las grandes mejoras y el progreso de la prevención en la salud oral, el desarrollo de caries dental, más específicamente la desmineralización del esmalte o manchas blancas, continúa siendo una consecuencia no deseada del tratamiento de ortodoncia ^24^. Los diferentes aparatos y materiales de ortodoncia dificultan las medidas de higiene bucal y, consecuentemente, incrementan el riesgo de LMB ^20^. De allí la importancia de la presente revisión de la literatura que presenta evidencia científica existente sobre el tema. 

La incidencia de LMB está estrechamente relacionada a la técnica de tratamiento de ortodoncia utilizada, y es mayor en ortodoncia con aparatología fija y menor en ortodoncia con alineadores. La menor incidencia de LMB con alineadores podría atribuirse a la posibilidad de realizar una mejor higiene oral, ya que el paciente puede retirarse el alineador para comer y cepillarse los dientes; por ende, tendría niveles de placa más bajos y un mejor índice de higiene oral ^26^. 

Por otro lado, un tiempo de tratamiento mayor es directamente proporcional a una mayor probabilidad de desarrollar LMB. Esta observación está respaldada por los resultados del trabajo de Ritcher *et al*. ^37^ quienes demostraron que, por cada mes de tratamiento con *multibrackets*, el número de LMB aumentaba en aproximadamente 0,08 lesiones por mes.

Respecto de la incidencia según el método de diagnóstico utilizado, los estudios revelan variaciones considerables en la incidencia de LMB durante el tratamiento de ortodoncia, lo que se debería a diferencias en los métodos para evaluar y clasificar las descalcificaciones ^24^. Los estudios que evaluaron las LMB por medio del análisis de fluorescencia inducida por luz encontraron más lesiones que aquellos que realizaron el diagnóstico a través de la inspección visual. Esto puede deberse a que, al usar la FIL, las lesiones de manchas blancas pueden detectarse de forma temprana, además de ser un método de detección confiable y consistente en el tiempo ^25^. Por otra parte, cabe señalar que el uso de fotografías intraorales para la determinación de LMB en pacientes de ortodoncia está aumentando y siendo estandarizado, ya que este sistema digital tiene varias ventajas: los registros son permanentes, pueden ser examinados más tarde y también reexaminados varias veces, las fotografías se pueden digitalizar y clasificar independientemente por varios examinadores, y la gravedad de la lesión se puede cuantificar midiendo la intensidad de colores; sin embargo, factores como la iluminación y la angulación generan dudas con respecto a la fiabilidad de éste método ^20^.

Una mayor incidencia de LMB fue hallada en la zona del tercio gingival en caninos e incisivos laterales maxilares. Estos resultados sugieren que las áreas de superficies dentales pequeñas que quedan a menudo entre el bracket y la encía son propicias para la retención de placa. Además, las ansas de cierre y los ganchos, a menudo, son colocados en el arco entre los incisivos laterales y caninos, lo que dificulta la higiene en la zona vestibular. Sumado a esto, la concavidad mesiogingival del incisivo lateral superior puede generar un vacío entre la base del *bracket* y la superficie dental, lo que a su vez puede causar retención de placa y caries ^22^. 

Finalmente, uno de los estudios revisados ^23^ menciona que existe una regresión de la LMB durante el primer año después del retiro de la aparatología. Este evento, podría tener lugar debido a la exposición continua de los dientes a saliva después del descementado de los *brackets* de ortodoncia que permite la recreación de un equilibrio fisiológico mediante procesos naturales de remineralización; por lo tanto, la desmineralización podría detenerse y, a veces, corregirse si la lesión afecta sólo a las capas externas del esmalte ^24^.

## CONCLUSIONES

El tratamiento de ortodoncia está asociado a una mayor prevalencia e incidencia de LMB. La edad y el sexo del paciente no incrementan de forma significativa su incidencia durante el tratamiento de ortodoncia. La mayor incidencia se presenta en la zona del tercio gingival en caninos e incisivos laterales maxilares. Asimismo, la incidencia de LMB está estrechamente relacionada con la técnica de tratamiento de ortodoncia utilizada siendo mayor en ortodoncia con ligado convencional, seguida de ortodoncia con autoligado y menor en ortodoncia con alineadores. Además, un tiempo de tratamiento mayor es directamente proporcional a una mayor probabilidad de desarrollar LMB. Existe una regresión de las LMB durante el primer año después del retiro de la aparatología donde la desmineralización puede detenerse, e incluso corregirse si la lesión solo afecta a las capas más externas del esmalte.

## Fuente de financiamiento:

Autofinanciado
